# Herbicide Resistance Traits in Maize and Soybean: Current Status and Future Outlook

**DOI:** 10.3390/plants8090337

**Published:** 2019-09-09

**Authors:** Vijay K. Nandula

**Affiliations:** Crop Production Systems Research Unit, Agricultural Research Service, United States Department of Agriculture, Stoneville, MS 38776, USA; vijay.nandula@usda.gov

**Keywords:** corn, herbicide resistance trait, maize, soybean

## Abstract

This article reviews, focusing on maize and soybean, previous efforts to develop nontransgenic herbicide-resistant crops (HRCs), currently available transgenic HRC traits and technologies, as well as future chemical weed management options over the horizon. Since the mid twentieth century, herbicides rapidly replaced all other means of weed management. Overreliance on ‘herbicide-only’ weed control strategies hastened evolution of HR weed species. Glyphosate-resistant (GR) crop technology revolutionized weed management in agronomic crops, but GR weeds, led by Palmer amaranth, severely reduced returns from various cropping systems and affected the bottom line of growers across the world. An additional problem was the lack of commercialization of a new herbicide mode of action since the 1990s. Auxinic HRCs offer a short-term alternative for management of GR Palmer amaranth and other weed species. New HRCs stacked with multiple herbicide resistance traits and at least two new herbicide modes of action expected to be available in the mid-2020s provide new chemical options for weed management in row crops in the next decade.

## 1. Introduction

Weeds cause extensive losses amounting to billions of US$ [1] through increased production costs, decreased quality and quantity of produce, reduced aesthetic value of landscapes that they thrive in, health effects on humans and pets, and other undesirable effects such as fuel for forest fires, etc. Over the past several centuries, weeds have been controlled with mechanical, biological, and cultural tools. Chemical weed control with inorganic compounds was extensively practiced in the late-nineteenth to mid-twentieth century, with earliest evidence even pointing back to the Roman era [2]. The real ‘Chemical Era’ of weed control started in the 1940s with the discovery of 2,4-dichorophenoxyacetic acid (2,4-D) during World War II chemical warfare research [2]. Since then, several herbicides belonging to different chemical classes and possessing diverse modes of action have been synthesized and commercialized around the world. Herbicides rapidly replaced all other means of weed management due to their superior efficacy, relatively low cost, selectivity, and targeted weed control. There has been at least one herbicide labeled for every cropping system imagined. Herbicides provided advantages such as increased productivity, improved quality of produce, reduced drudgery of hand weeding, and reduced soil erosion and top soil loss due to reduced cultivation and tillage (enhanced by less fossil fuel use). Overreliance on herbicides alone pushed weed species toward evolving resistance to herbicides. The astronomical cost of commercializing a new herbicide active ingredient (cost of discovery, development, and regulatory approval of a new synthetic pesticide was estimated to be $280 million in 2016 [3] coupled with the paucity of new herbicide modes of action [3] steered the agrochemical industry toward engineering/development of crops resistant to ‘currently’ available (subject to change) herbicides. This review covers maize (*Zea mays* L.) and soybean (*Glycine max* (L.) Merr.) only with discussion of earlier efforts to develop herbicide-resistant crops (HRCs), currently available HRC technologies, and future developments in the HRC arena.

## 2. Early Efforts

HRCs can be classified as nontransgenic (traditional genetic methods of selection of resistance traits) and transgenic (genetically engineered). Nontransgenic HRCs were developed using conventional breeding techniques (Table 1) such as seed mutagenesis (soybean resistant to sulfonylurea herbicides, disclosed in 1987), pollen mutagenesis (maize resistant to imidazolinone herbicides, released in 1992), and mutations in tissue culture (maize resistant to imidazolinone herbicides, revealed in 1991; to sethoxydim, an acetyl-CoA carboxylase (ACCase) inhibitor, released in 1992; soybean resistant to metribuzin, a triazine herbicide, disclosed in 1996) [4,5]. Agronomic performance of nontransgenic HRCs met with modest acceptance in the marketplace and often did not reach the expectations of growers and commodity groups. Scientists began to look at alternative ways to develop HRCs as weed management tools, to manage a broad spectrum of weeds, with superior agronomic characteristics.

## 3. Current Transgenic HRCs

Currently commercialized transgenic HRCs (some with associated herbicide formulations registered by the US Environmental Protection Agency (EPA)) are summarized in Table 2. The following sections describe these HRCs separated by crop and herbicide mode of action. The context and issues pertaining to each resistant trait are limited to the geographical region where they have been commercialized. For example, glyphosate-resistant (GR) traits and related aspects are common across North America (Canada and US) and South America (Brazil and Argentina), whereas dicamba-resistant crop technologies are restricted to the US.

## 4. Maize

The real breakthrough occurred in the 1990s with the commercial release of glyphosate-resistant (GR) crops. These crops allowed the application of glyphosate multiple times in the growing season without the risk of crop injury. Glyphosate was, hitherto, used nonselectively for weed control in vineyards, orchards, rights-of-way, industrial areas, and railroads. It has been deemed as “a once-in-a-century herbicide” [7] for its broad weed spectrum, reasonable cost, favorable environmental properties, and association with the widely popular GR crops. In susceptible plants, glyphosate inhibits 5-enolpyruvylshikimate-3-phosphate synthase (EPSPS), a key enzyme in the shikimate pathway responsible for the biosynthesis of aromatic amino acids and several secondary metabolites in the phenylpropanoid pathway.

GR maize was introduced in 1998 [5,6]. Transformation of maize plants with *CP4* (an *Agrobacterium* species strain) *EPSPS* and e35S promoter produced plants with vegetative resistance to glyphosate but reduced male fertility [8]. Therefore, the first-generation GR maize, trademarked as Roundup Ready^®^ (RR) trait, GA21 utilized the rice actin 1 promoter driving the gene for a GR form of maize EPSPS (*TIPS-EPSPS*) [8], (*ZM-EPSPS*) [5,6]. A new event NK603, with two copies of a slightly modified *EPSPS CP4* gene, was developed to improve maize tolerance to glyphosate at both vegetative and reproductive stages, and was commercially released in 2001 in a breeding stack with glufosinate and four insect resistance traits [5].

Glufosinate inhibits the enzyme glutamine synthetase (GS), which catalyzes assimilation of ammonia with glutamate to form glutamine [8]. Glufosinate resistance is due to metabolic inactivation by an acetyltransferase enzyme that catalyzes the acetylation of glufosinate [5]. Two glufosinate resistance genes, *bar* and *pat*, encode homologous enzymes [9]. Both genes were isolated from soil microorganisms, *pat* from *Streptomyces viridochromogenes* and *bar* from *Streptomyces hygroscopicus* [6]. Glufosinate-resistant maize was commercialized for the first time in 1996 stacked with *Bt* insect resistance, as a stand-alone trait in 1997 [6], and was combined with GR maize as a ‘double stacked trait’ in the mid-2000s.

2,4-D is an auxin herbicide with phytotoxic action limited to broadleaf weed species. However, 2,4-D-resistant maize was developed in tandem with 2,4-D-resistant soybean, trademarked as Enlist™ Weed Control System by Corteva Agriscience (process described in a later section) and deregulated by the US Department of Agriculture (USDA) and an associated low volatility 2,4-D choline formulation registered by EPA for use only in 2,4-D-resistant crops, both in 2014, but not commercially launched in the US until 2018. It is to be noted that 2,4-D had been labeled for use in maize over the past several decades, both as preemergence and postemergence applications. A type of aryloxyalkanoate dioxygenase (AAD) enzyme was identified that provided resistance to 2,4-D as well as a class of ACCase inhibiting herbicides, popularly known as ‘fops’ belonging to the aryloxyphenoxypropionate (AOPP) chemical family, for example, quizalofop [10,11]. 2,4-D and the ‘fop’ herbicides possess an identical bond that facilitates their metabolism by a common enzyme. Due to concerns that GR grass weeds would run amuck before commercialization of 2,4-D-resistant crops, 2,4-D-resistant maize was promoted to control GR grass. Another class of ACCase inhibiting herbicides, the ‘dims’ belonging to the cyclohexanedione chemical family, lack the above bond and can be used to manage volunteer 2,4-D-resistant maize. Invariably, most transgenic maize HRCs on the market also carry insect-resistance traits (*Bt* trait), which will not be discussed here.

## 5. Soybean

In 1996, GR soybean was the first GR crop to be commercialized. The first generation of GR soybean, event 40-3-2, were the most successful outcome of over-expressing the glyphosate-insensitive *CP4 EPSPS* in all tissues using strong, constitutive viral promoters such as e35S or FMV from cauliflower or figwort mosaic viruses, respectively [8]. The first-generation GR soybean went off patent in 2015, which means individuals can grow them and save seed for re-use as long as the seed has no other trait or varietal patents [12]. Although the first-generation GR soybean has been phased out of the seed stock (of formerly Monsanto Co., now Bayer Crop Science), some institutions in Missouri and Arkansas have done breeding with this older trait and developed cultivars exhibiting the trait.

The second generation of GR soybean was commercialized in 2009 with a broader launch in 2010 as Roundup Ready 2 Yield^®^ (RR2Y) by Monsanto Co. [8]. Several seed companies still sell RR2Y cultivars and they were available in 2019 [12]. The RR2Y event, MON89788 contained the same *CP4 EPSPS* as GTS 40-3-2, but with the gene inserted at a different site in an elite variety “A3244” with a different promoter and regulatory elements to enhance expression in the sensitive tissues [5].

Glufosinate-resistant soybean, with the *pat* gene and the CaMV 35S promoter, was publicly released for sale in 2009 as a promising tool to combat GR weeds, especially tall water hemp (*Amaranthus tuberculatus* Moq. Sauer) and Palmer amaranth (*Amaranthus palmeri* S. Watson) [5]. The glufosinate-resistant trait in soybean has been a good candidate for stacking in other herbicide-resistant soybean cultivars (discussed in a later section).

From the 2019 seed sales season, the glufosinate resistance technology now rests in the hands of BASF Crop Protection, who purchased it from Bayer Crop Science as part of anti-trust remediation [12]. Glufosinate-resistant crops including soybean have been steadily gaining market share as GR weeds spread across the southern and midwestern US, approaching 20% of the soybean market share. Low seed prices coupled with availability of generic glufosinate herbicides make this technology, labeled as LibertyLink System^®^, a viable option for soybean growers and is available on BASF’s Credenz^®^ soybean platform and other independent seed companies totaling 78 licensees [12].

Dicamba-resistant soybean, Roundup Ready 2 Xtend^®^ (RR2Xtend) from formerly Monsanto Co., now Bayer Crop Science, was deregulated by the Animal and Plant Health Inspection Service (APHIS) of the USDA in 2015. Dicamba monooxygenase (DMO), from the soil bacterium *Pseudomonas maltophilia* (strain DI-6), encodes for Rieske nonheme monooxygenase that converts dicamba to 3-6-dichlorosalicylic acid (DCSA) [13]. The genetically engineered version of the *DMO* gene for expression in higher plants used the FLt36 promoter from peanut chlorotic streak virus, a translational enhancer from the tobacco etch virus (TEV), a chloroplast transit peptide–coding region from the pea Rubisco small subunit gene for chloroplast localization of DMO, and a terminator region from the pea Rubisco small subunit gene (*rbcS3′*) [13].

**Figure 1:**
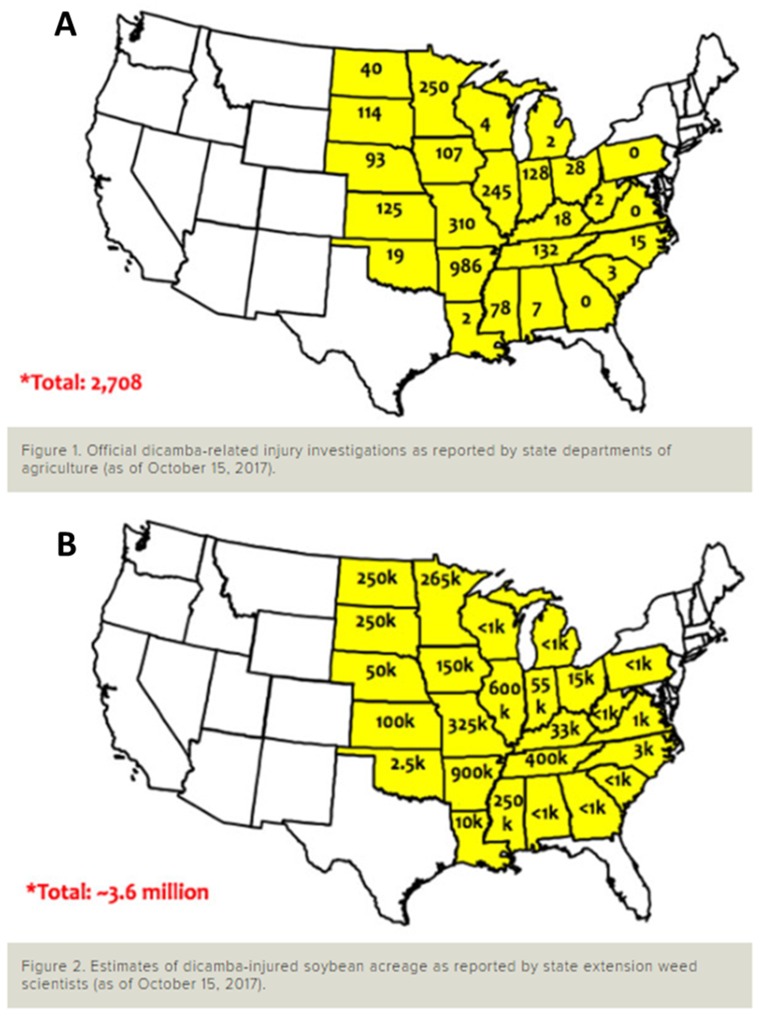
Official dicamba-related injury investigations as reported by state departments of agriculture (**A**) and estimates of dicamba-injured soybean acreage as reported by state extension weed scientists (**B**) in the 2017 growing season in the US.

Formulations of dicamba specifically labeled for use in RR2Xtend were not registered until fall 2016. Three dicamba formulations, XtendiMax^®^ and FeXapan^®^, both containing the diglycolamine (DGA) salt of dicamba, and Engenia^®^ comprising the BAPMA (N, N-Bis-(aminopropyl) methylamine) salt of dicamba were registered for use in the US by EPA in 2016 until 2018; in 2018 registration was extended until December 2020. In 2017, additional restrictions were implemented toward application of the above formulations which were labeled for use only in dicamba-resistant crops. In 2019, Tavium^®^ containing dicamba DGA salt plus *S*-metolachlor was registered by EPA. In 2016, illegal/off-target/off-label applications of dicamba via formulations other than XtendiMax^®^, FeXapan^®^, and Engenia^®^ were made on dicamba-resistant soybean and cotton (*Gossypium hirsutum* L.) in AR, MO, TN, MS, and several other states, resulting in injury to non-dicamba-resistant crops and sensitive flora across the landscape from dicamba drift (volatile/vapor drift and/or physical drift due to droplet movement owing to temperature inversion and other factors). The issue of injury to non-dicamba-resistant crops from dicamba drift was compounded multifold in 2017 when registered dicamba applications in dicamba-resistant soybean were made over large swaths of the cropping area. Dicamba had been labeled for use in maize over the past several decades, both as preemergence and postemergence applications. However, dicamba has not been applied in the middle of the growing season, when temperatures are usually higher than during preplant or early crop growing conditions, or when several sensitive plant species are present, prior to commercialization of dicamba-resistant crops. In the 2017 growing season, a total of 1.44 million ha of dicamba-injured soybean were estimated from 2708 official dicamba-related injury investigations as reported by individual state departments of agriculture and state extension weed scientists in the US (Figure 1) [14]. It was believed, by several row-crop production practitioners, that the soybean hectarage reported above is a gross underestimation. In 2018, there were fewer complaints of soybean injury compared to 2017, probably, due to more growers planting dicamba-resistant soybean as an insurance against injury, neighboring growers settling disputes off the record, a marked improvement in efficiency of applications, or a combination of more than one of the above reasons. Records of injury from dicamba drift in 2018 and 2019 are available elsewhere in the literature.

The RR2Xtend crop hectarage estimates ranged from 16.2 to 20.2 million ha in 2018 [12]. The RR2Xtend soybean trait was available from a variety of seed companies via licensing agreements in 2019. It allows growers to spray dicamba and glyphosate postemergence to the crop.

Another group of auxin HRCs are the 2,4-D- resistant crop technologies, developed by formerly Dow AgroSciences and MS Technologies, now managed by Corteva Agriscience. Several species of bacteria possess families of *tfdA* genes that are known to produce 2,4-D-metabolizing enzymes [15,16]. A *tfdA* transgene, originally isolated from the bacterium *Alcaligenes eutrophus* [17], conferred resistance to 2,4-D when expressed in cotton. This gene, independently discovered in bacteria isolated from soil exposed to 2,4-D [18], increased tolerance in grapes (*Vitis vinifera* L.) to 2,4-D by 100-fold. 

The *aad-1* gene isolated from a gram-negative soil bacteria, *Sphingobium herbicidovorans*, codes for a Fe(II) and 2-ketoglutarate-dependent dioxygenase that degrades the alkanoate side chains of both 2,4-D and members of the AOPP class of ACCase inhibitors to a hydroxyl [10]. Another gene sequence called *aad-12*, isolated from *Delftia acidovorans*, codes for a 2-ketoglutarate-dependent dioxygenase that inactivates phenoxyacetate auxins such as 2,4-D and pyridinyloxyacetate auxins such as triclopyr or fluroxypyr, but not commercial AOPPs [11]. The 2,4-D resistance traits were coded DHT1 for maize and DHT2 for soybean [19].

2,4-D-resistant soybean, trademarked as Enlist E3™, contain a single molecular stack providing resistance to glufosinate and glyphosate were commercialized in 2019. The original Enlist soybean with 2,4-D and glyphosate resistance was deregulated in 2014 but was not planted due to international import limitations. Another type of Enlist soybean, Enlist Soy + RR2Y, also confers tolerance to these herbicides, but Corteva Agriscience and MS Technologies focused their 2019 commercial offerings on Enlist E3 soybean in the US, Canada, and Brazil after international import restrictions were lifted by China and Philippines in early 2019 [12,20]. In 2018, some farmers in Indiana, Illinois, and Ohio planted Enlist E3 soybean within a stewarded closed-loop system based on an agreement between then Dow AgroSciences and Archer Daniels Midland (ADM) Company, as well as seed production acres. Corteva Agriscience is working with more than 100 independent seed companies to broadly license the Enlist E3 soybean trait, which will enable wide availability for 2020 and beyond [20].

2,4-D formulations labeled for use with the Enlist Weed Control System include Enlist™ Duo (2,4-D choline salt + dimethylammonium salt of glyphosate) and Enlist™ One (2,4-D choline salt) with COLEX-D™ Technology. 2,4-D products that do not contain COLEX-D™ Technology are not authorized for use in conjunction with Enlist crops.

Herbicides that inhibit the enzyme 4-hydroxyphenylpyruvate dioxygenase (HPPD) represent the last group of commercialized chemical weed control products with a unique mode of action. A new herbicide resistance technology, designated as GT27™ Soybean Performance System, originally developed by Bayer Crop Science and associated entities, confers resistance to glyphosate and a new HPPD-inhibiting herbicide isoxaflutole (ALITE 27, previously Balance Bean) applied preemergence only, pending EPA registration as of July 2019. The trait was commercially available in 2019 as a standalone product offered by some independent seed companies. However, BASF, which now owns the LibertyLink^®^ technology, has made this technology available in 2019 as LibertyLink^®^ GT27™ stack through its Credenz^®^ soybean platform (maturity groups from 0 to 4.5) providing the option to use glyphosate, ALITE 27, and glufosinate. The major risk is that growers will be drawn to applying cheaper generic HPPD inhibitors on the LL GT27 soybean, inviting crop injury.

MGI soybean, carrying resistance to mesotrione, glufosinate, and isoxaflutole, were codeveloped by BASF and Syngenta [21]. The MGI soybean trait, that received approval from China, is scheduled for commercial launch in 2020, contingent on all regulatory approvals. 

## 6. Future HRCs and Related Technologies

Although the USDA deregulated the dicamba resistance trait in 2016, dicamba-resistant maize, trademarked as XtendFlex^®^ maize by Bayer has not been commercialized. Bayer has applied to the EPA regarding expanding uses of its XtendiMax dicamba formulation to dicamba resistant maize. Dicamba-resistant maize, in a three-way stack with glyphosate and glufosinate resistance, could be in the market in the next few years and is projected to be planted on 32 million ha or 89% of the US maize area [22]. Similarly, dicamba-resistant soybean and cotton exceeded expectations with a combined area of 24 million ha in 2019 as against initial estimates of 16 to 16.8 million ha [22].

A choline formulation of dicamba, to be applied only in RR2Xtend crops, is under development, but has not been registered with the EPA for commercialization yet.

Escalating costs of discovery and development of new herbicide active ingredients with new modes of action have resulted in a drought for these much-needed tools required to manage both wild-type and herbicide-resistant weed populations around the world. A January 2019 announcement by the FMC Corporation was welcome news [23]. The company will introduce two new herbicide modes of action over the next decade. An unnamed active molecule from a chemical group has been targeted for rice (*Oryza sativa* L.) and could be commercialized in five years. Other molecules from this group will be developed for maize and soybean. Another molecule, yet un-named, with another mode of action and targeting Palmer amaranth in maize and soybean will be launched, approximately, in 2026. 

It is hoped and anticipated that additional herbicide modes of action will be announced by research and discovery groups in major agrochemical companies in the next few years. For example, Bayer Crop Science announced the potential for a new maize and soybean herbicide mode of action in the late 2020s [24].

## 7. Worldwide Use of Transgenic Crops

The data discussed in this section have been gleaned from the website of the International Service for the Acquisition of Agri-Biotech Applications [25]. Transgenic crop area in 2017 attained new record-high adoption at 189.8 million ha worldwide—an increase of 4.7 million ha (11.6 million A) or 3% from 185.1 million ha in 2016. The average transgenic crop adoption rate reached ≥93% or close to saturation in the US, Canada, Brazil, Argentina, and India. The global area of transgenic crops has increased ~112-fold from 1.7 million ha in 1996 to 189.8 million ha in 2017 making transgenic crops the fastest adopted crop technology. An accumulated 2.3 billion ha was achieved in 22 years (1996–2017) of transgenic crop commercialization. A total of 67 countries adopted transgenic crops, with 24 countries planting and 43 additional countries importing transgenic produce.

Transgenic soybean covered 50% of the global transgenic crop area, occupying 94.1 million ha, and corn was second with 59.7 million ha. The area planted with transgenic crops with stacked traits (insect resistance and herbicide resistance) increased by 3% and occupied 41% of the global transgenic crop area and only herbicide resistance traits were planted to 47% of the global area.

It is most likely that the approvals for transgenic crops in the countries that are members of the European Union (EU) are not for growing in most of the EU countries but for importation of the harvested crops for animal feed. The number of approvals of HRC events is summarized in Table 3. 

## 8. Conclusions

Chemical weed control will continue to remain the dominant method of weed management in the short term, despite the evolution of GR and other HR weeds, including 2,4-D and dicamba-resistant Palmer amaranth and tall water hemp [26,27]. New maize, soybean, and other crop technologies, providing resistance to multiple herbicides and improved formulations, and new herbicide modes of action (expected in the next five to ten years) offer additional tools to growers toward effective and sustainable weed management. Public and private land managers must implement nonchemical control strategies such as cultural, biological, and mechanical means wherever and whenever possible to sustain the rapidly depleting herbicide portfolio as well as to prepare for new patterns of weed emergence, growth, and resistance evolution in response to prevailing weather conditions. Pending an unforeseen revolutionary technology, chemical weed control will be necessary to meet current and future challenges of weed management.

## Figures and Tables

**Table 1:** Nontransgenic herbicide-resistant maize and soybean.

Selection Method	Herbicide Family	Crop	Year of Disclosure
Seed mutagenesis	Sulfonylurea	Soybean	1987
Pollen mutagenesis	Imidazolinone	Maize	1992
Tissue culture	ACCase inhibitor	Maize	1992
	Imidazolinone	Maize	1991
	Triazine	Soybean	1996

Adapted from [4,5]. ACCase, acetyl-CoA carboxylase.

**Table 2:** Current transgenic herbicide-resistant maize and soybean and associated trait genes.

Crop	Resistance Trait	Trait Gene	Trait Designation	First Sales	Trade Name
Maize	Glyphosate	Three modified maize *epsps*	GA21	1998	Roundup Ready^®^
		Two *cp4 epsps*	NK603	2001	Roundup Ready^®^ 2
	Glufosinate	*pat*	T14, T25	1996	LibertyLink System^®^
	2,4-D	*tfdA*	DHT1	2019	Enlist™ Weed Control System
	AOPP	*aad*	DHT1	2019	Enlist™ Weed Control System
Soybean	Glyphosate	*cp4 epsps*	GTS 40-3-2	1996	Roundup Ready^®^
		*cp4 epsps*	MON89788	2009	Roundup Ready^®^ 2 Yield
	Glufosinate	*pat*	A2704-12	2009	LibertyLink System^®^
	Dicamba	*dmo*	MON87708	2017	Roundup Ready 2 Xtend^®^
	2,4-D	*tfdA*	DHT2	2019	Enlist™ Weed Control System

Partly adapted from [5,6]. AOPP: Aryloxyphenoxypropionate; 2,4-D: 2,4-Dichorophenoxyacetic acid.

**Table 3:** Global approvals of herbicide-resistant maize and soybean transgenic events [25].

Event	Crop	# of approvals
NK603	Maize	55 approvals in 26 non-EU countries plus 28 EU countries
GTS 40-3-2	Soybean	54 approvals in 27 non-EU countries plus 28 EU countries
GA21	Maize	50 approvals in 24 non-EU countries plus 28 EU countries
A2704-12	Soybean	43 approvals in 23 non-EU countries plus 28 EU countries
T25	Maize	41 approvals in 20 non-EU countries plus 28 EU countries
